# The CIMP-high phenotype is associated with energy metabolism alterations in colon adenocarcinoma

**DOI:** 10.1186/s12881-019-0771-5

**Published:** 2019-04-09

**Authors:** Maria S. Fedorova, George S. Krasnov, Elena N. Lukyanova, Andrew R. Zaretsky, Alexey A. Dmitriev, Nataliya V. Melnikova, Alexey A. Moskalev, Sergey L. Kharitonov, Elena A. Pudova, Zulfiya G. Guvatova, Anastasiya A. Kobelyatskaya, Irina A. Ishina, Elena N. Slavnova, Anastasia V. Lipatova, Maria A. Chernichenko, Dmitry V. Sidorov, Anatoly Y. Popov, Marina V. Kiseleva, Andrey D. Kaprin, Anastasiya V. Snezhkina, Anna V. Kudryavtseva

**Affiliations:** 1 Engelhardt Institute of Molecular Biology, Russian Academy of Sciences, Moscow, Russia; 2 National Medical Research Radiological Center, Ministry of Health of the Russian Federation, Moscow, Russia; 3 State Hospital №57, Moscow, Russia

**Keywords:** Colon adenocarcinoma, CIMP, RNA-Seq, TCGA, Energy metabolism

## Abstract

**Background:**

CpG island methylator phenotype (CIMP) is found in 15–20% of malignant colorectal tumors and is characterized by strong CpG hypermethylation over the genome. The molecular mechanisms of this phenomenon are not still fully understood. The development of CIMP is followed by global gene expression alterations and metabolic changes. In particular, CIMP-low colon adenocarcinoma (COAD), predominantly corresponded to consensus molecular subtype 3 (CMS3, “Metabolic”) subgroup according to COAD molecular classification, is associated with elevated expression of genes participating in metabolic pathways.

**Methods:**

We performed bioinformatics analysis of RNA-Seq data from The Cancer Genome Atlas (TCGA) project for CIMP-high and non-CIMP COAD samples with DESeq2, clusterProfiler, and topGO R packages. Obtained results were validated on a set of fourteen COAD samples with matched morphologically normal tissues using quantitative PCR (qPCR).

**Results:**

Upregulation of multiple genes involved in glycolysis and related processes (*ENO2, PFKP, HK3, PKM, ENO1, HK2, PGAM1, GAPDH, ALDOA, GPI, TPI1,* and *HK1*) was revealed in CIMP-high tumors compared to non-CIMP ones. Most remarkably, the expression of the *PKLR* gene, encoding for pyruvate kinase participating in gluconeogenesis, was decreased approximately 20-fold. Up to 8-fold decrease in the expression of *OGDHL* gene involved in tricarboxylic acid (TCA) cycle was observed in CIMP-high tumors. Using qPCR, we confirmed the increase (4-fold) in the *ENO2* expression and decrease (2-fold) in the *OGDHL* mRNA level on a set of COAD samples.

**Conclusions:**

We demonstrated the association between CIMP-high status and the energy metabolism changes at the transcriptomic level in colorectal adenocarcinoma against the background of immune pathway activation. Differential methylation of at least nine CpG sites in *OGDHL* promoter region as well as decreased *OGDHL* mRNA level can potentially serve as an additional biomarker of the CIMP-high status in COAD.

**Electronic supplementary material:**

The online version of this article (10.1186/s12881-019-0771-5) contains supplementary material, which is available to authorized users.

## Introduction

Colon adenocarcinoma (COAD) is a common and socially significant disease, nevertheless, some mechanisms of its onset and progression have been studied insufficiently [1–3]. The advent of high-throughput “omics” methods has made it possible to detect alterations that occur during the malignant transformation and progression of COAD. These include changes in methylation patterns, mutation spectra, non-coding RNA profiles, cell signaling, as well as metabolic pathways at both mRNA and protein levels [1, 2, 4, 5]. “Omics” data are useful for molecular classification of malignant tumors, drug discovery, and developing personalized approach to optimize colon adenocarcinoma management.

Traditionally, colorectal cancer (CRC) is characterized with several molecular features, these are: the presence or absence of microsatellite instability (MSI), chromosomal instability (CIN), CpG island methylator phenotype (CIMP), and spectrum of the mutations in the driver genes. The accumulation of multidimensional "omics" data on colorectal cancer made it possible to link these features and to develop consensus molecular classification of CRC. As a result of complex analysis of six independent classification systems, four consensus molecular subtypes (CMS) with distinguishing features were identified: CMS1 (“MSI/Immune”, 14%), CMS2 (“Canonical”, 37%), CMS3 (“Metabolic”, 13%), and CMS4 (“Mesenchymal”, 23%) [6].

Like other malignant neoplasms, COAD is characterized by the alteration of various cell signaling and metabolic pathways, including energy metabolism [7–11]. Mitochondrial dysfunction and activation of glycolysis is a well-known hallmark of cancer [12–15]. The activation of glycolysis is commonly accompanied with increased expression of hexokinases, which are the enzymes that participate in the first stage of glycolysis. This provides an opportunity to search for therapeutic targets among genes involved in cell energy metabolism [16, 17]. However, disturbances in gene expression, signaling and metabolic pathways can be different depending on particular driver alterations (e.g. mutations in RAS-RAF cascade). Previously, we found that expression of hexokinases was upregulated at the mRNA level in only 10–15% of COAD samples [18]. Furthermore, it has been shown that increased expression of *HK1* and *HK2* in COAD tissue is associated with an unfavorable prognosis [19, 20].

Unsupervised clustering of the promoter DNA methylation profiles revealed four methylation clusters. Two of them contained tumors with elevated methylation rates and belonged to so called CpG island methylator phenotype (CIMP, CIMP+, and CIMP-positive) discovered in CRC by Toyota et al. [21, 22]. CIMP phenotype occurs in 15–20% of CRC samples and characterized by excessive methylation levels predominantly in promoter regions across the genome. CIMP-positive group can be divided into two subtypes, CIMP-high (CIMP-H) and CIMP-low (CIMP-L), according to the level of hypermethylation and some other features. A CIMP-high subgroup exhibits an exceedingly high frequency of genome hypermethylation; it is strongly associated with *MLH1* promoter hypermethylation and with the presence of *BRAF* V600E mutation. A CIMP-low subgroup is enriched for *KRAS* mutations and characterized by DNA hypermethylation of a subset of CIMP-high associated markers as well as a unique group of CpG sites [1, 23]. Nevertheless, genome-wide methylation profiling revealed at least two more clusters containing non-CIMP samples. Both clusters predominantly included tumors that originated from different locations [1, 23]. Unfortunately, neither mRNA-Seq data nor miRNA profiling can help to differentiate these two non-CIMP clusters. Comparison of methylation clustering analysis with other quantitative “omics” data identified significantly overlapping only for one group: one out of three mRNA clusters partially corresponded to CIMP-high tumors, it was also enriched with hypermutated tumors. The absence of clear criteria defining CIMP-positive and non-CIMP tumors, especially their more detailed classification, makes them difficult to be identified in routine laboratory practice. Currently there are several approaches for the identification of CIMP-positive samples [24–26].

In terms of novel consensus classification, CIMP-positive COAD samples are mostly included in CMS1 and CMS3 subgroups. CMS3 is characterized by metabolic dysregulation and contains predominantly CIMP-low tumors. The most common alterations in CMS1 subgroup, which is mostly comprised of CIMP-high samples, are hypermutations, MSI, and strong immune activation. It is assumed that CIMP-high status is associated with MSI through the methylation of *MLH1* promoter regions. Being inactivated, *MLH1,* a participant of the mismatch repair system, does not prevent the accumulation of DNA damage. However, the particular mechanisms of association between energy metabolic changes and CIMP status are still unclear [27].

Therefore it is expected that the development of CIMP status is a part of more complex process. This involves specific driver mutations in the genes such as *KRAS, BRAF*, *IDH1*, and *IDH2,* the activation of glycolysis and general metabolic changes, as well as deregulation in many other cell signaling pathways [28–30]. Moreover, in some malignant tumors, the driver mutations in the genes encoding “metabolic” enzymes, such as IDH1, SDHx, and others, are sufficient to further establish methylator phenotype [31–33]. To summarize, the hypermethylation of cancer cell genome is considered to be commonly associated with the alterations in energy metabolism [27]. Nevertheless, strong metabolic alterations (e.g. energy metabolism shift) are postulated only for CMS3 subgroup mostly including CIMP-low samples with *KRAS* mutations [6]. Remarkably, CMS1 subgroup, predominantly represented by CIMP-high tumors, is not referred to be characterized with the metabolic alterations, first of all, by the energy metabolism shift. This fact does not play into the current understanding of the carcinogenesis mechanisms and empirically established common rules and tendencies in different tumor types.

In the present work, we analyzed the association between CIMP-high status and alterations of energy metabolism in colon adenocarcinoma using RNA-Seq data from The Cancer Genome Atlas (TCGA) project. Obtained results were verified with quantitative PCR (qPCR) on a set of COAD samples derived from Russian patients. We revealed deregulation in the expression of several genes involved in the energy metabolism as well as activation of immune-associated pathways in CIMP-high colorectal adenocarcinoma.

## Materials and methods

### Bioinformatics analysis

We performed differential gene expression analysis of TCGA RNA-Seq data (colon adenocarcinoma dataset). We focused on the patients of the Caucasian race (163 samples), identified in the database as “white”, as they most closely genetically resemble the Russian population. We carried out two comparisons: between pools of tumor and normal tissues, and between CIMP-high and non-CIMP tumor samples. CIMP status was included in the annotation provided by TCGA consortium [1].

Differentially expressed genes (DEGs) were identified using the DESeq2 Bioconductor package [34]. We selected top DEGs that passed DESeq2 FDR < 0.05 and Mann-Whitney *p* < 0.05 thresholds. Next, we performed over-representation (OR) and gene set enrichment (GSEA) tests for KEGG pathways and top DEGs using the clusterProfiler package [35]. Then, we visualized differential expression profiles of genes involved in several KEGG pathways, including “Glycolysis/Gluconeogenesis” and “TCA cycle”, using the pathview Bioconductor package [36]. Some KEGG nodes represent a set of proteins with similar function or isozymes. For example, lactate dehydrogenase (LDH) corresponds both to LDHA and LDHB. This means that the sum of bidirectional gene expression changes within the same KEGG node will be visualized as the retention in mRNA level. Therefore, we edited the automatically generated diagrams to reveal the expression alterations of each gene within the KEGG nodes if its differentially expressed.

For detailed analysis of epigenetic characteristics in *OGDHL* promoter region across TCGA cohort, we compared CIMP-high and non-CIMP tumors in context of differential CpG methylation. TCGA dataset derived with Illumina Infinium HumanMethylation450 microarrays (17 CpG sites per gene, on the average) was used. Unfortunately, the original TCGA research paper [1] published in 2012 contains annotation on CIMP status only for samples analyzed with the previous generation of methylation-sensitive microarrays, HumanMethylation27 (only 1 CpG site per gene, on the average). Hence, we developed an approach of identifying CIMP-like samples based on genome-wide methylation profiling data. First, we selected CpG sites located in gene promoter regions using ENCODE genomic segments annotation (consensus of ChromHMM and SegWay methods; 110,000 of 300,000 CpG sites passed this filter). Next, for each sample we calculated *M*_*A*_, an average of 80th, 85th, 90th, and 95th percentiles (we used the average value in order to reduce stochastic noise) of β-values across passed CpG sites (β-value is the ratio of methylated alleles for a current CpG). The derived *M*_*A*_ values indicated the overall hypermethylation level for each sample. According to *M*_*A*_ distribution, all the samples were divided into three groups: “non-CIMP”, “CIMP-low”, and “CIMP-high”.

As expected, CIMP-high status (*versus* non-CIMP) demonstrated statistically significant Spearman (anti)correlations with MSI-high (*r* = 0.53), hypermutation phenotype (*r* = 0.49), mutations in *BRAF* (*r* = 0.46), *APC* (*r* = − 0.27), and *p53* (*r* = − 0.24) that perfectly agrees with the observations made for common CIMP-high samples, which had been selected regarding to annotation in TCGA consortium paper [1].

### Tissue specimens

In total, fourteen COAD samples (seven CIMP-high samples and seven non-CIMP ones) and matched morphologically normal tissues were obtained after surgical resection prior to radiation or chemotherapy. The samples were frozen and stored in liquid nitrogen. Morphological classification of the tumors was performed according to the WHO Classification of Tumors of the Digestive System [37]. Only samples comprised of ≥70% tumor cells were included in the analysis. Written informed consent was obtained from all patients. The study was approved by The Ethics committee of Herzen Moscow Cancer Research Institute, a branch of the National Medical Research Radiological Center, Ministry of Health of the Russian Federation and was performed in accordance with the principles outlined in the Declaration of Helsinki (1964). Information on the patients and characteristics of the COAD tumors, including age, gender, grade, TNM staging, *KRAS*, *NRAS*, and *BRAF* mutations, microsatellite instability (MSI), and CIMP status, were determined (Table 1).

**Table 1:** Clinicopathological and molecular genetic characteristics of the COAD patients.

Patient	Age	Gender	Grade	TNM stage	*KRAS* status	*NRAS* status	*BRAF* status	MSI status	Relative mRNA level		
									*ENO2*	*OGDHL*	*PKLR*
CIMP-high COAD patients											
Pat1	63	F	2	T3N1M0	WT	WT	c. 1799 T → A (p.Val600Glu)	MSI-H	25↓	1.9↑	1.1↑
Pat2	76	M	2	T3N0M0	WT	WT	c. 1799 T → A (p.Val600Glu)	MSI-H	25↓	2.6↑	2.1↑
Pat3	69	F	2	T3N0M0	WT	WT	c. 1799 T → A (p.Val600Glu)	MSI-H	25↓	4.0↑	2.4↑
Pat4	62	F	2	T3N1M0	WT	WT	c. 1799 T → A (p.Val600Glu)	MSI-H	20↓	2.0↑	1.4↑
Pat5	77	F	3	T2N0M0	WT	WT	c. 1799 T → A (p.Val600Glu)	MSI-H	1.1↓	1.1↑	1.3↓
Pat6	55	F	2	T3N0M0	WT	WT	c. 1799 T → A (p.Val600Glu)	MSS	4.3↑	2.8↓	1.2↑
Pat7	76	F	2	T3N1M0	WT	WT	WT	MSS	2.5↓	1.7↑	2.4↑
Non-CIMP COAD patients											
Pat8	85	M	2	T3N0M0	WT	WT	WT	MSS	1.1↓	3.5↑	1.1↓
Pat9	83	M	2	T3N0M0	WT	WT	WT	MSS	7.4↑	1.7↑	5.1↑
Pat10	67	F	2	T3N0M0	WT	WT	WT	MSS	3.2↑	1.0	5.7↑
Pat11	77	F	2	T3N0M0	WT	WT	WT	MSS	24.8↑	14.3↓	10.2↑
Pat12	59	F	3	T3N1M0	WT	WT	WT	MSS	5.7↑	4.8↓	1.7↓
Pat13	69	F	2	T4N1M0	WT	WT	WT	MSS	3.8↑	4.8↓	7.7↑
Pat14	55	F	2	T3N0M0	WT	WT	WT	MSS	30.0↑	2.9↓	13.3↑

*Note*: ↑ - expression increase; ↓ - expression decrease.

### Nucleic acid isolation and cDNA synthesis

Genomic DNA and total RNA were extracted from the fresh-frozen tissue. DNA was isolated using a MagNA Pure Compact Nucleic Acid Isolation Kit I - Large Volume (Roche, Switzerland). RNA was isolated with a MagNA Pure Compact RNA Isolation Kit (Roche). DNA and RNA quantification was performed on a Quibit 2.0 fluorometer (Thermo Fisher Scientific, USA). The RNA integrity number was measured using an Agilent Bioanalyzer 2100 (Agilent Technologies, USA). cDNA synthesis was performed using M-MLV Reverse Transcriptase (Thermo Fisher Scientific) and random hexamers.

### Microsatellite instability testing and identification of mutation status in *KRAS*, *NRAS,* and *BRAF* genes

Sanger sequencing was performed for *KRAS* (exons 2, 3, and 4), *NRAS* (exons 2, 3, and 4), and *BRAF* (exon 15) [38]. MSI status was analyzed using five common markers (D2S123, D5S346, D17S250, BAT25, and BAT26). MSI-high (MSI-H) status was defined as instability in two or more of the five markers, and MSI-low (MSI-L) status was detected as instability in only one of the five markers [39]. The distinction between microsatellite stable (MSS) and MSI-low can only be accomplished if a greater number of markers is estimated. A unique clinical and pathological phenotype is identified for the MSI-high tumors (approximately 15% of malignant colorectal tumors). Tumors with MSI-low and MSS status appear to be phenotypically similar.

### CIMP status testing

The first step of this analysis was represented by sodium bisulfite conversion of genomic DNA (1 μg) using a Zymo Research EZ DNA Methylation Kit (Zymo Research, USA) with a final eluted volume of 20 μl. The converted DNA was diluted as 1:10 for MethyLight analysis, and the methylated and unmethylated controls (CpGenome Universal Methylated/Unmethylated DNA, Millipore, USA) were diluted as 1:80. A total of 5 μl of diluted DNA was used per PCR reaction. Additionally, serial dilutions of the methylated DNA control were run in each PCR plate for standard curve generation.

Testing for CIMP status was performed using methyl-specific qPCR for eight markers localized in promoter regions of the *CDKN2A* (NM_000077.4 transcript, traditionally called *p16*), *CACNA1G*, *IGF2*, *NEUROG1*, *RUNX3*, *SOCS1*, *CRABP1*, and *hMLH1* genes. Ogino et al. suggested using these eight markers for the routine laboratory stratification of colorectal cancer samples. Depending on amount of positive markers, samples were divided into three groups: CIMP-high, CIMP-low, and non-CIMP. A sample is considered to be CIMP-low if one to five out of eight markers are methylated, and to be CIMP-high if six to eight markers are methylated. The promoter regions of *ACTB* and *COL2A1* genes were used for normalization [40, 41].

### Quantitative PCR

Gene expression levels were estimated by qPCR. Amplification was performed in triplicates using TaqMan assays (Thermo Fisher Scientific): *OGDHL* (Hs00971806_m1), *ENO2* (Hs00157360_m1), and *PKLR* (Hs00176075_m1) on the ABI PRISM® 7500 Sequence Detection System (Thermo Fisher Scientific) following the manufacturer’s instructions. *RPN1* and *GUSB* were used as reference [42, 43] genes. The PCR program was as follows: 10 min at 95 °C, then 50 two-step cycles of 15 s at 95 °C and 60 s at 60 °C. The total reaction volume was 20 μL.

The Relative Quantitation software (Thermo Fisher Scientific) and “Analysis of Transcription of Genes” tool were used to analyze the qPCR data considering the efficiency of the PCR amplification. Relative expression levels of the target genes were calculated using the ΔΔCt method [11, 44, 45].

To analyze the differences in relative (tumor-to-normal) gene expression levels between groups of CIMP-high and non-CIMP samples, we applied the nonparametric Mann-Whitney U-test and considered *p* < 0.05 as statistical significant event.

## Results

### Bioinformatics analysis

First, we performed the comparison between pools of COAD and normal samples based on TCGA RNA-Seq data. The analysis revealed the altered expression of glycolytic genes (a part of “Glycolysis/Gluconeogenesis” KEGG pathway) occurring along with the downregulation of many genes that participate in the tricarboxylic acid (TCA) cycle (a part of “TCA cycle” KEGG pathway). Together, these results suggest that the activation of glycolysis and suppression of mitochondrial respiration may occur in COAD (Additional files 1, 2, 3, and 4).

Decreased expression was observed for only a few glycolytic genes, including *HK2* (2-fold). Increased expression was revealed in a significantly larger number of glycolytic genes, including *PFKM, ALDOC*, *LDHA, LDHB, PKM, PGK1*, *ENO3*, *GAPDH, PGK1*, *ENO1*, and *GPI* (1.4-1.8-fold). As expected, the gluconeogenesis was predominantly downregulated at the transcription level and was characterized by decreased expression of *PCK1* (8-fold) and *G6PC* (3-fold) genes. It is worth noting that we report only average changes in gene expression level between the two groups. Within each group, the expression level values are heterogeneous and take on various values. Due to the large sampling size, we were able to observe even slight trends toward up- or downregulation between the groups (e.g. 1.2-1.5-fold). These differences were statistically significant (Mann-Whitney test, *p* < 0.05).

In contrast to glycolytic genes, the expression of genes involved in the TCA cycle and related processes was mostly characterized by decrease: *SUCLG2* (3-fold); *SDHD, PC*, *IDH3A*, and *ACO2* (2-fold); *MDH1*, *SUCLG1*, and *IDH1* (1.5-fold). The increased expression (1.5-fold) was noticed for only three genes participating in TCA cycle - *ACLY*, *IDH2*, and *MDH2*.

Next, we tested the hypothesis whether the disturbance of energy metabolism is related to increased genome methylation level (CIMP-high) and is not limited by “Metabolic” molecular subtype (CMS3) according to consensus classification [6]. Bioinformatics analysis of TCGA data revealed the increased expression of the glycolytic genes in CIMP-high COAD samples compared to non-CIMP ones. The same cohort represented in TCGA database was used for this comparison as for described above. It is important that CIMP status description was already included in the annotation provided by TCGA consortium. These results are presented in the Additional files 5, 6, 7, and 8.

Most remarkably, the expression of the *PKLR* gene, encoding for pyruvate kinase involved in gluconeogenesis, was decreased approximately 20-fold. In all CIMP-high samples, the expression of *PKLR* was extremely low (only few reads per sample). Increased expression (1.5-3.0-fold) was observed for *ENO2*, *PFKP, HK3*, *PKM, ENO1*, *HK2*, *PGAM1*, and *GAPDH* genes. A less marked increase in expression (approximately 1.2-fold) was observed for *ALDOA*, *GPI*, *TPI1*, and *HK1*. Among the genes encoding TCA cycle enzymes, we observed that the expression of *OGDHL* was decreased 8-fold. Like *PKLR* gene, the *OGDHL* expression was very weak in 95% of CIMP-high samples. Several genes showed a slight increase in the expression, in particular, mRNA level of *SDHA* and *DLST* was increased 1.5-fold, while *CS* expression was increased 1.2-fold. It should be noted that in CIMP-high tumors we observed significant upregulation of dozens of genes involved in the immune response including chemokines, cytokines and many others that are predominantly expressed by immune cells.

### Validation of the gene expression in Russian patients

Based on the results of bioinformatics analysis, we selected several DEGs involved in energy metabolism (*ENO2*, *PKLR*, and *OGDHL)* for experimental validation with qPCR in CIMP-high and non-CIMP COAD samples. Clinicopathological and molecular genetic characteristics of COAD samples are represented in Table 1.

**Fig. 1:**
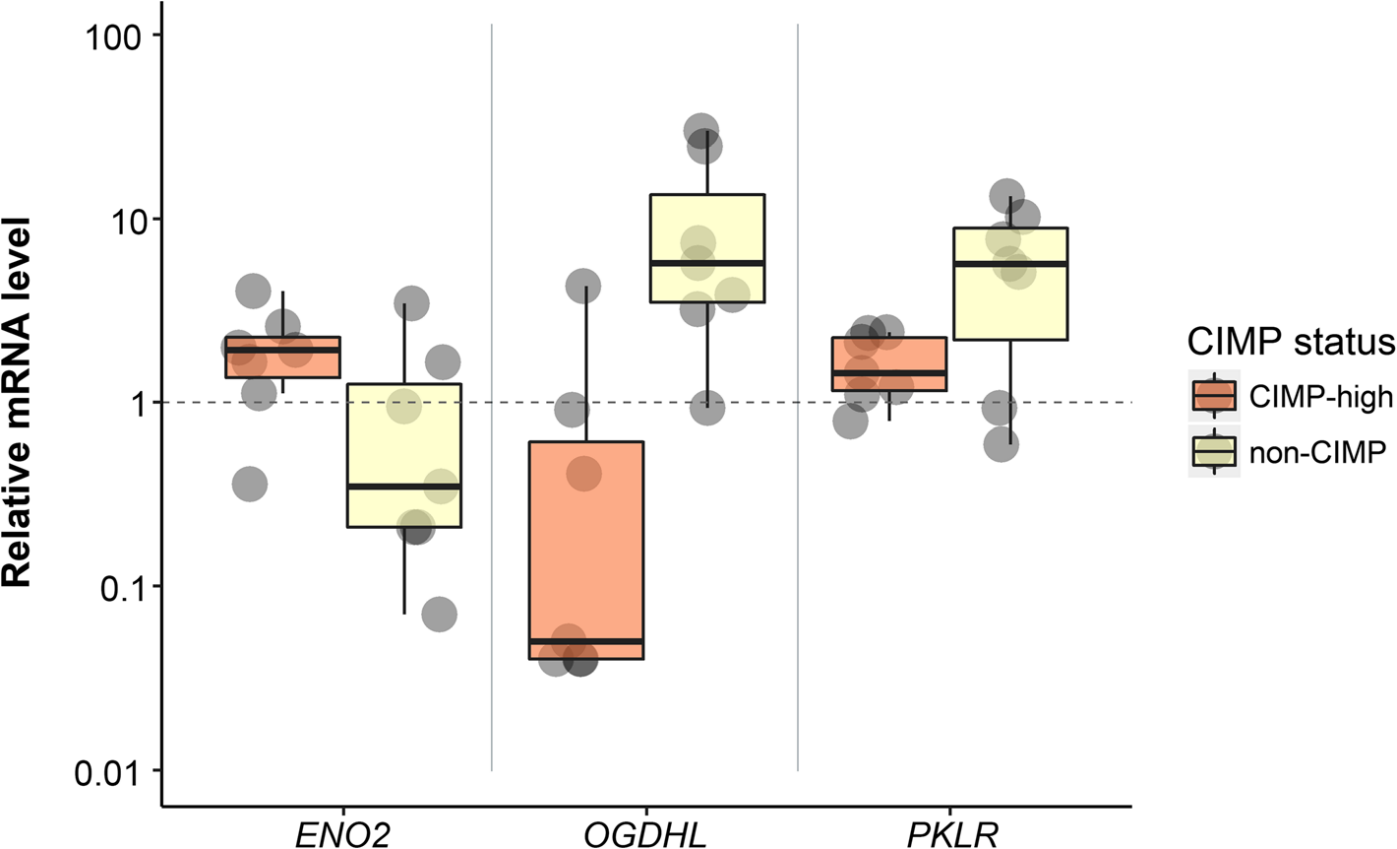
Boxplots illustrating relative mRNA levels (tumor *versus* matched normal tissue) of *ENO2*, *OGDHL*, and *PKLR* genes in two groups of COAD samples (with CIMP-high and non-CIMP status) according to qPCR data. The boxes show quartile range (25th-75th percentiles). The central lines in boxes show a median value. Among these genes, *OGDHL* and *ENO2* differential expression is statistically significant between CIMP-high and non-CIMP tumors (*p* < 0.05)

Downregulation of *OGDHL* expression (*p* = 0.007) was observed in CIMP-high COAD samples (Tables 1,2, Fig. 1). Statistically significant (*p* = 0.049) upregulation of *ENO2* mRNA level was also found in CIMP-high group. Additionally, it was obseved a tendency for *PKLR* downregulation, nevertheless, *p*-value was not statistically significant (*p* = 0.165).

**Table 2:** Expression of *ENO2*, *PKLR*, and *OGDHL* genes in CIMP-high and non-CIMP COAD samples

	CIMP-high samples			Non-CIMP samples		
Genes	Frequency of mRNA level changes, %		Median of mRNA level changes, n-fold	Frequency of mRNA level changes, %		Median of mRNA level changes, n-fold
	↑	↓		↑	↓	
*ENO2*	57 (4/7)	14 (1/7)	1.2↑	14 (1/7)	57 (4/7)	1.0
*OGDHL*	14 (1/7)	71 (5/7)	2.0↓	85 (6/7)	0 (0/7)	10.8↑
*PKLR*	43 (3/7)	0 (0/7)	1.6↑	71 (5/7)	14 (1/7)	6.2↑

### Analysis of *OGDHL* promoter region methylation

We found a significant decrease in *OGDHL* mRNA level in CIMP-high COAD samples compared to non-CIMP ones. As the aberrant methylation of promoter region is one of the most frequent mechanisms of expression decrease, we assessed if this does contribute to *OGDHL* downregulation in COAD. CpG methylation status was analysed based on TCGA data derived with Illumina Infinium HumanMethylation450 microarrays. Whereas these samples didn’t have information about CIMP status in the TCGA annotation, we used the developed approach (see Materials and Methods) of identifying “non-CIMP”, “CIMP-low”, and “CIMP-high” groups based on methylation profiling with the microarrays.

**Fig. 2:**
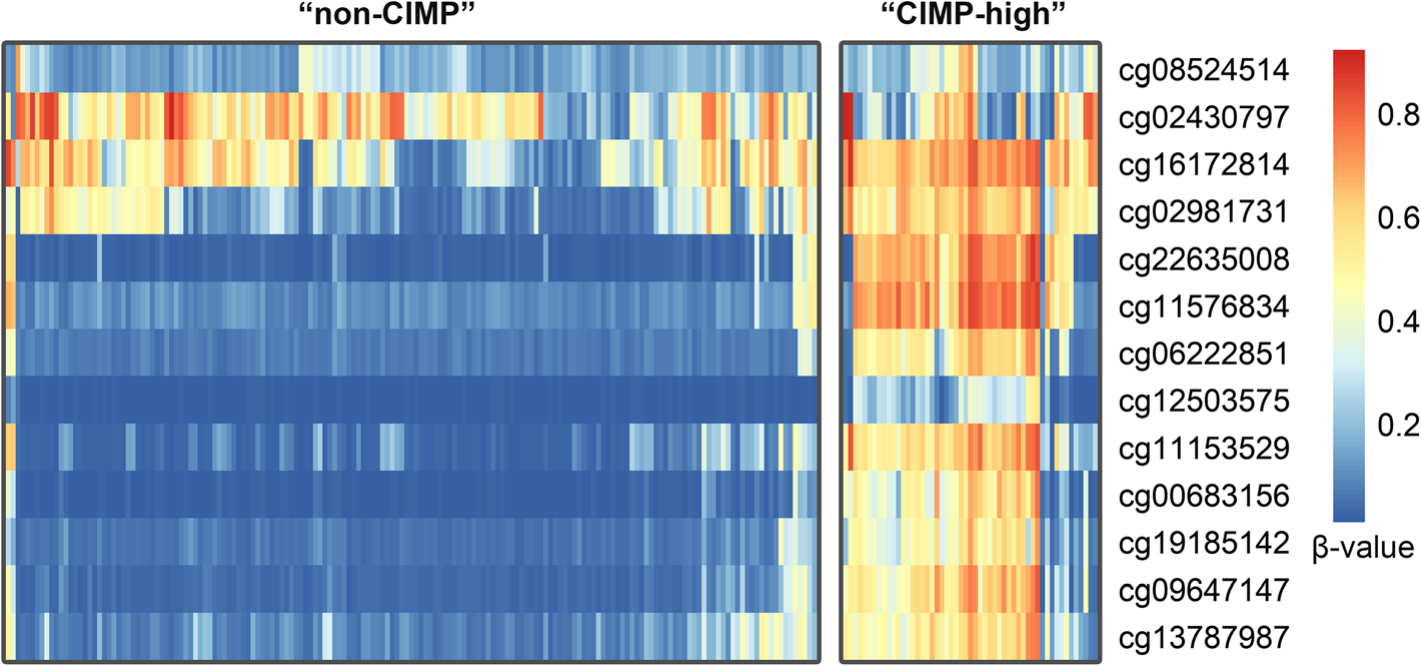
Heatmap illustrating distribution of beta-values of 13 CpG sites in the promoter region of *OGDHL* gene in “CIMP-high” and “non-CIMP” samples

For at least nine CpG sites in *OGDHL* promoter region, we found a differential methylation between “non-CIMP” and “CIMP-high” samples (Fig. 2). This fact demonstrates that some loci could serve as additional markers of high methylation level across the genome.

## Discussion

Glycolysis and TCA cycle are interconnected to various cell signaling and metabolic pathways that are important in the context of carcinogenesis and found to be frequently disturbed in tumors: fatty acid and lipid biosynthesis, synthesis of amino acids and nucleotides, stabilization and degradation of HIF1-a, and others. Many genes involved in glycolysis and TCA cycle are considered to be prognostic markers for different cancer types. In the present work, we found a clear trend towards upregulation of glycolytic genes in colon adenocarcinoma, especially CIMP-high tumors. Indeed, increased glycolytic rate, a hallmark of cancer cells, represents not only a mechanism of adaptation to hypoxic conditions but provide a source of ATP and building material that met needs of intensively proliferating cells [46]. We focused on two KEGG pathways, hsa00010 (“Glycolysis/Gluconeogenesis”) and hsa00020 (“TCA cycle”). Despite the names, these KEGG pathways partially intersect to each other and contain additional metabolic branches.

### “Glycolysis/gluconeogenesis” KEGG pathway

Cancer cells are characterized by activation of glucose catabolism together with mitochondrial dysfunction and commonly inactivated glucose synthesis. Thus, ATP and the intermediate products required for active growth and proliferation are produced intensively [47, 48]. We found expression upregulation of ten genes (1.5–3-fold) and downregulation of three genes (1.3–2-fold) participating in glycolytic process.

The first stage of this process is the phosphorylation of glucose catalyzes by hexokinases (HK1, HK2, and HK3). Traditionally, it has been believed that the expression level of hexokinases, especially *HK2*, is increased in colorectal cancer [49–51]. However, we have previously demonstrated a decrease in the expression of *HK1* and *HK2* in majority of colorectal cancer samples [18].

In the present study, we observed decreased *HK2* expression, and upregulation of *HK3* expression in COAD. In hepatocellular carcinoma, overexpression of hexokinase 2 is associated with *HK2* CpG island hypermethylation and hypomethylation of surrounding promoter regions. These methylation state changes can serve as a prognostic marker [52]. The *HK2* promoter region contains binding sites for AP-1 transcription factors, as well for the Myc proto-oncogene and mutant forms of p53 [53]. Mutant forms of p53 activate *HK2* expression, and can account for the relationship between the loss of cell cycle control in rapidly growing tumors and activation of glycolysis. High levels of *HK2* expression were observed in the invasive margins of colorectal tumors [20]. In combination with decreased p-PDH expression, *HK2* overexpression may represent a marker of unfavorable prognosis, tumor aggressiveness, and recurrence in patients with colorectal cancer [54].

*HK3* is expressed at high levels in the bone marrow and spleen. Previously, we have shown a frequent and significant increase in *HK3* expression (more than 100-fold) in colorectal cancer [55]. However, the absolute number of *HK3* transcripts in cells is significantly lower than that of the other hexokinases.

We also found 1.5-fold (on the average) upregulation of *GAPDH*, encoding for glyceraldehyde-3-phosphate dehydrogenase, a key glycolytic enzyme that is present in all tissues and has multiple functions [56, 57]. *GAPDH* is traditionally referred as an endogenous control gene for qPCR, however, its increased expression has been reported for various malignant tumors [58–64]. *GAPDH* plays an important role in the formation of malignant tumor phenotypes and is considered as a promising target for therapy [16, 17].

Glycolysis rate is regulated both at gene expression level and allosterically (enzymes can be inhibited with their products). It can be assumed that the observed overexpression of *PFKM*, *ALDOC*, *GAPDH*, and *ENO1* contribute to the activation of glycolysis. For several tumor types, the main activator of glycolysis is PFKM, and inhibition of this enzyme significantly reduces the growth and invasion of tumor cells [65, 66]. Increased *ALDOB* and *ALDOC* expression can lead to Wnt-signaling pathway activation in tumor cells, which contributes to disease progression [67]. However, in gastric cancer and hepatocellular carcinoma, the opposite effect has been demonstrated, and decreased *ALDOB* expression was found to be associated with poor prognosis [68, 69].

Increased *ENO1* expression is observed in many types of malignant tumors and is associated with aggressive phenotypes [70–72]. Enolase is considered a promising target for anti-cancer therapy [73].

Upregulation of *PGK1* is associated with the rapid progression and metastasis of stomach carcinoma [74, 75], as well as with the progression of breast, prostate, pancreatic, and ovarian cancers [76–79]. Inhibition of PGK1 may also represent a method of antitumor therapy [74, 80].

Traditionally, lactate dehydrogenase is thought to be associated with the progression and aggressiveness of tumors, as lactate accumulation causes microenvironmental acidification, destroys the intercellular matrix, and enhances metastatic spread [81]. Hypermethylation of the *LDHB* promoter and decreased gene expression are observed in various tumor cell lines. These events may be associated with intensive proliferation, migration, and invasion [82, 83]. In contrast, in triple-negative breast cancer cells, increased *LDHB* expression is observed, and associations between *LHDB* overexpression and unfavorable prognosis were demonstrated. However, despite contradictory results, *LDHB* is an important participant in carcinogenesis and is considered as a potential target for anti-cancer therapy [84].

We revealed activation of some glycolytic genes as well as suppression of genes involved in gluconeogenesis including *G6PC* and *PCK1.* The G6PC enzyme catalyzes the synthesis of glucose from glucose-6-phosphate, whereas *PCK1* encodes a key gluconeogenesis enzyme catalyzing the conversion of oxaloacetate to phosphoenolpyruvate. Downregulation of *G6PC* gene probably leads to the accumulation of glucose-6-phosphate, the excess of which can be converted to ribose-5-phosphate and used for the synthesis of nucleotides [85, 86].

We found the significant activation of *ALDH3B2* expression in COAD samples. This gene encodes one of the isozymes of aldehyde dehydrogenase. It is possible that the product of the *ALDH3B2* gene could serve as the enzyme protecting tumor colon cells from ROS (reactive oxygen species), considering that expression of other alcohol dehydrogenase isozymes (*ADH1B* and *ADH1C*) is decreased [14].

### “TCA cycle” KEGG pathway

We found decreased expression of a number of genes involved in the TCA cycle and related processes. Decreased expression of aconitase, encoded by *ACO1* and *ACO2*, is described for many types of tumor cells, and represents an unfavorable prognostic marker in gastric cancer [87]. The decreased activity of these enzymes increases the amount of citrate in the nucleus, which can then be sent to the cytosol. Excessive citrate is usually converted to acetyl-CoA, which is actively used by tumor cells for de novo fatty acid biosynthesis. Elevated citrate lyase (*ACLY*) expression indicates active acetyl-CoA synthesis. A characteristic feature of various types of malignant tumors is the rapid growth of tumor biomass and the related activation of lipid biosynthesis [88]. Suppression of *ACLY* expression reduces the population of cancer stem cells in many cell lines with a wide range of genetic differences [89].

Decreased expression of genes encoding subunits of succinate dehydrogenase (*SDHx*) can result in the accumulation of succinate, which helps to stabilize HIF1-a [90]. The HIF1-a transcription factor regulates many genes involved in carcinogenesis and tumor angiogenesis [91]. Disturbances in the expression of oncogenes and tumor growth suppressor genes, such as *p53* and *HIF1-a*, can directly or indirectly affect the expression of various components of the TCA cycle [92].

In many types of tumor cells, the degree of TCA cycle productivity is related to glutamine enzyme activity, which directs glutamine to the cycle in the form of alpha-ketoglutarate [93], explaining the extreme dependence of certain types of tumors on glutamine [94]. Tumor cells need to maintain a high level of TCA cycle metabolite biosynthesis, including that of alpha-ketoglutarate and isocitrate, which can be transported to the cytoplasm to participate in the synthesis of nucleotides and a number of amino acids [95, 96]. Increased expression of *IDH2*, encoding for a mitochondrial form of NADP^+^-dependent isocitrate dehydrogenase, is associated with this. The IDH2 enzyme is able to perform both direct and reverse reactions, converting isocitrate to alpha-ketoglutarate and vice versa. Thus, deficiency of any of these important intermediates can be compensated. NAD^+^-dependent isocitrate dehydrogenase (*IDH3*) and its isozymes catalyze the TCA cycle limiting step and their role in the development of malignant tumors has been actively investigated [97–99]. Only the IDH3 enzyme only catalyzes a direct reaction, but it is allosterically regulated and adjusts activity to the needs of the cell [100].

### Association of CIMP phenotype with alterations of energy metabolism

The existence of tumors characterized by simultaneous hypermethylation of a large number of CpG islands was described in colorectal cancer and called the CpG island methylator phenotype [3]. This molecular phenotype occurs in 15–20% of malignant colorectal tumors [101]. Development of CIMP-positive tumors is associated with elderly age, female gender, higher degree of differentiation and mucinous histological type, localization in the proximal colon, mutations in *KRAS* or *BRAF*, and wild-type *TP53* [25, 102]. The mechanisms, through which aberrant DNA hypermethylation is induced in CIMP-positive tumors, are not absolutely clear, but there several factors may be associated with this process. These include metabolic shift towards energy production via glycolysis or the development of more pronounced Warburg effect. Our study revealed CIMP-high associated changes in the expression of genes involved in energy metabolism, including participants of glycolysis, TCA cycle, as well as related processes. For CIMP-low COAD, metabolic alterations were reported in different samplings [6].

Methylation pattern as well as mutational status and other genetic characteristics allowed creating a consensus molecular classification system consisting of four robust subtypes with clear biological interpretability [6]. Distinct differences in the intrinsic biological underpinnings of each molecular group provide the new taxonomy of this disease. Important associations were found between CMS subgroups and clinical variables, as well as prognosis. CMS1 tumors were frequently diagnosed in females with right-sided tumors with higher grade and had very poor survival after relapse. It is likely that this phenomenon is associated with the presence of samples with MSI-high status and *BRAF*-mutations as the main part of CMS1 subgroup. CMS2 tumors were mainly left-sided and had superior survival after relapse. CMS4 tumors tended to be diagnosed at more advanced stages; they are more aggressive and displayed worse overall survival [6].

The analysis of TCGA RNA-Seq data in CIMP-high *versus* non-CIMP COAD samples revealed altered expression levels of many genes involved in energy metabolism, including *ENO2*, *PKLR*, and *OGDHL*. Alterations observed in *ENO2* and *OGDHL* genes were validated by qPCR.

Enolase is a glycolytic enzyme that catalyzes the conversion of 2-phosphoglycerate into phosphoenolpyruvate. In mammalian tissues, enolase is represented by three tissue-specific isozymes [103, 104]. Enolase 2 (*ENO2*) is induced by hypoxia, which typically occurs in tumors. Along with other glycolytic genes, *ENO2* was previously found to be overexpressed in colorectal cancer and other cancer types [105–107]. *ENO2* (alpha-enolase) is significantly upregulated in a metastatic colon cancer cell line, suggesting a possible association with the metastatic process in vitro and in vivo [108]. Moreover, *ENO2* is a prognostic factor of small cell lung, breast, and prostate cancer [109–112]. qPCR analysis revealed statistically significant differences in *ENO2* expression in CIMP-high and non-CIMP COAD samples (*p* = 0.049).

Pyruvate kinase, PKLR, is highly expressed in liver tissues and red blood cells. Analysis of *PKLR* expression revealed its association with metastases. PKLR promotes colon cancer cell metastases to the liver, but does not promote basal cell growth in culture [113]. PKLR provides cell survival in the central parts of a tumor, where the cells are in conditions of high density and hypoxia. Under these conditions, PKLR is required to maintain levels of the major endogenous antioxidant, a glutathione, and support cancer cell survival [113]. Our results show that the average mRNA level of *PKLR* in CIMP-high tumors is approximately 5-fold lower than that in non-CIMP COAD samples. However Mann-Whitney test have not been demonstrated statistically significant *p*-value for this gene (*p* = 0.165).

*OGDHL* expression demonstrated the most striking differences between CIMP-high and non-CIMP samples, based on both TCGA and qPCR data. This gene encodes a subunit of the oxoglutarate dehydrogenase complex, which is involved in the TCA cycle as well as in the induction of apoptosis. Various pathways and factors causing apoptosis can be classified as either extrinsic or intrinsic. The extrinsic pathway begins from the activation of apoptosis-associated receptors by various ligands, including Fas and TNF. Intrinsic apoptotic pathways are induced by DNA damage, oxidative stress, and other factors [114]. Mitochondria play a central role in both cases. The mitochondrial intermembrane space contains a number of pro-apoptotic proteins including cytochrome c (Cyto c), endonuclease G, and apoptosis-associated factor (AIF). Bax/Bak are involved in the formation of the mitochondrial outer membrane permeabilization pore, which leads to the release of cytochrome c and other proteins into the cytoplasm [115, 116]. In turn, cytochrome c causes oligomerization of the Apaf-1 and further assembly of the apoptosome, which is responsible for cleavage of pro-caspase 9, a key step in apoptosis [16, 115, 117]. Mitochondria and the glutarate dehydrogenase complex in particular, are the main source of free radicals within the cell [118]. It was shown that OGDHL, and its close homolog OGDH, are localized in the mitochondria, and that overexpression of *OGDHL* results in increased free radical content and lipid peroxidation [119]. In present work a significant decrease was found in *OGDHL* expression in CIMP-high COAD samples compared to non-CIMP ones. And at least nine CpG sites have sufficiently different methylation pattern in these two groups. Probably these particular CpG sites along with decrease expression level could serve as additional markers of high methylation level across the genome.

Therefore, we have demonstrated that COAD samples characterized by CIMP-high status (mostly represented with CMS1 group) carry metabolic alterations together with activation of immune-related pathways. Partially, these changes could be explained by the presence of transcripts originated from the tumor infiltrating lymphocytes.

## Conclusion

We have revealed that the energy metabolic changes manifested in the overexpression of glycolytic genes along with the downregulation of genes participating in TCA cycle are associated with the CIMP-high status in COAD. This results are consistent with the conception assuming that hypermethylation of cancer genome is correlated with the increased glycolytic rate. At the same time, we also found significant activation of the genes involved in immune-related pathways in CIMP-high COAD samples, which is a hallmark of the "Immune" subtype (CMS1) and represents an important prognostic factor of colorectal cancer. Probably, all common CRC molecular characteristics are intertwined and occur in several CMS subtypes.

## Additional files

Additional file 1: **Figure S1.** Differential expression profiles of “Glycolysis/Gluconeogenesis” KEGG pathway genes in colon adenocarcinomas compared to normal tissues (TCGA data). The color scale reflects the base-2 logarithm of the ratio of the gene expression level in tumor tissues to the expression level in normal tissues. Red color indicates upregulated genes, green - downregulated. The scale is relative; minimal and maximal values in the color scale are corresponding to the greatest decreased or increased expression level among the all analyzed genes. (PDF 5115 kb)

Additional file 2: **Table S1.** Differential expression profiles of “Glycolysis/Gluconeogenesis” KEGG pathway genes in colon adenocarcinomas compared to normal tissues (TCGA data). The color scale indicates the degree of change. (XLSX 836 kb)

Additional file 3: **Figure S2.** Differential expression profiles of “TCA cycle” KEGG pathway genes in colon adenocarcinomas compared to normal tissues (TCGA data). The color scale reflects the base-2 logarithm of the ratio of the gene expression level in tumor tissues to the expression level in normal tissues. Red color indicates upregulated genes, green - downregulated. The scale is relative; minimal and maximal values in the color scale are corresponding to the greatest decreased or increased expression level among the all analyzed genes. (PDF 173 kb)

Additional file 4: **Table S2.** Differential expression profiles of “TCA cycle” KEGG pathway genes and transcripts in colon adenocarcinomas compared to normal tissues (TCGA data). The color scale indicates the degree of change. (XLSX 516 kb)

Additional file 5: **Figure S3.** Differential expression profiles of “Glycolysis/Gluconeogenesis” KEGG pathway genes in CIMP-high colon adenocarcinomas compared to non-CIMP ones (TCGA data). The color scale reflects the base-2 logarithm of the ratio of the gene expression level in CIMP-high tumors to the expression level in non-CIMP tumors. Red color indicates upregulated genes, green - downregulated. The scale is relative; minimal and maximal values in the color scale are corresponding to the greatest decreased or increased expression level among the all analyzed genes. (PDF 4613 kb)

Additional file 6: **Table S3.** Differential expression profiles of “Glycolysis/Gluconeogenesis” KEGG pathway genes in CIMP-high colon adenocarcinomas compared to non-CIMP ones (TCGA data). The color scale indicates the degree of change. (XLSX 266 kb)

Additional file 7: **Figure S4.** Differential expression profiles of “TCA cycle” KEGG pathway genes in CIMP-high colon adenocarcinomas compared to non-CIMP (TCGA data). The color scale reflects the base-2 logarithm of the ratio of the gene expression level in CIMP-high tumors to the expression level in non-CIMP tumors. Red color indicates upregulated genes, green - downregulated. The scale is relative; minimal and maximal values in the color scale are corresponding to the greatest decreased or increased expression level among the all analyzed genes. (PDF 928 kb)

Additional file 8: **Table S4.** Differential expression profiles of “TCA cycle” KEGG pathway genes in CIMP-high colon adenocarcinomas compared to non-CIMP tumors (TCGA data). The color scale indicates the degree of change. (XLSX 166 kb)

## Abbreviations

CIMP: CpG island methylator phenotype
COAD: Colon adenocarcinoma
GSEA: Gene set enrichment test
MSI: Microsatellite instability
TCGA: The Cancer Genome Atlas

## References

[1] Cancer Genome Atlas N (2012). Comprehensive molecular characterization of human colon and rectal cancer. Nature.

[2] Zhang B, Wang J, Wang X, Zhu J, Liu Q, Shi Z, Chambers MC, Zimmerman LJ, Shaddox KF, Kim S (2014). Proteogenomic characterization of human colon and rectal cancer. Nature.

[3] Kudryavtseva AV, Lipatova AV, Zaretsky AR, Moskalev AA, Fedorova MS, Rasskazova AS, Shibukhova GA, Snezhkina AV, Kaprin AD, Alekseev BY (2016). Important molecular genetic markers of colorectal cancer. Oncotarget.

[4] Krasnov GS, Dmitriev AA, Melnikova NV, Zaretsky AR, Nasedkina TV, Zasedatelev AS, Senchenko VN, Kudryavtseva AV (2016). CrossHub: a tool for multi-way analysis of the Cancer genome atlas (TCGA) in the context of gene expression regulation mechanisms. Nucleic Acids Res.

[5] Krasnov GS, Dmitriev AA, Kudryavtseva AV, Shargunov AV, Karpov DS, Uroshlev LA, Melnikova NV, Blinov VM, Poverennaya EV, Archakov AI (2015). PPLine: an automated pipeline for SNP, SAP, and splice variant detection in the context of Proteogenomics. J Proteome Res.

[6] Guinney J, Dienstmann R, Wang X, de Reynies A, Schlicker A, Soneson C, Marisa L, Roepman P, Nyamundanda G, Angelino P (2015). The consensus molecular subtypes of colorectal cancer. Nat Med.

[7] Kudryavtseva AV, Fedorova MS, Zhavoronkov A, Moskalev AA, Zasedatelev AS, Dmitriev AA, Sadritdinova AF, Karpova IY, Nyushko KM, Kalinin DV (2016). Effect of lentivirus-mediated shRNA inactivation of HK1, HK2, and HK3 genes in colorectal cancer and melanoma cells. BMC Genet.

[8] Snezhkina AV, Krasnov GS, Zaretsky AR, Zhavoronkov A, Nyushko KM, Moskalev AA, Karpova IY, Afremova AI, Lipatova AV, Kochetkov DV (2016). Differential expression of alternatively spliced transcripts related to energy metabolism in colorectal cancer. BMC Genomics.

[9] Fedorova M. S., Kudryavtseva A. V., Lakunina V. A., Snezhkina A. V., Volchenko N. N., Slavnova E. N., Danilova T. V., Sadritdinova A. F., Melnikova N. V., Belova A. A., Klimina K. M., Sidorov D. V., Alekseev B. Ya., Kaprin A. D., Dmitriev A. A., Krasnov G. S. (2015). Downregulation of OGDHL expression is associated with promoter hypermethylation in colorectal cancer. Molecular Biology.

[10] Snezhkina AV, Krasnov GS, Lipatova AV, Sadritdinova AF, Kardymon OL, Fedorova MS, Melnikova NV, Stepanov OA, Zaretsky AR, Kaprin AD (2016). The dysregulation of polyamine metabolism in colorectal Cancer is associated with overexpression of c-Myc and C/EBPbeta rather than Enterotoxigenic Bacteroides fragilis infection. Oxidative Med Cell Longev.

[11] Senchenko VN, Krasnov GS, Dmitriev AA, Kudryavtseva AV, Anedchenko EA, Braga EA, Pronina IV, Kondratieva TT, Ivanov SV, Zabarovsky ER (2011). Differential expression of CHL1 gene during development of major human cancers. PLoS One.

[12] Hanahan D, Weinberg RA (2011). Hallmarks of cancer: the next generation. Cell.

[13] Horne SD, Pollick SA, Heng HH (2015). Evolutionary mechanism unifies the hallmarks of cancer. Int J Cancer.

[14] Kudryavtseva AV, Krasnov GS, Dmitriev AA, Alekseev BY, Kardymon OL, Sadritdinova AF, Fedorova MS, Pokrovsky AV, Melnikova NV, Kaprin AD (2016). Mitochondrial dysfunction and oxidative stress in aging and cancer. Oncotarget.

[15] Oparina N. Yu., Snezhkina A. V., Sadritdinova A. F., Veselovskii V. A., Dmitriev A. A., Senchenko V. N., Mel’nikova N. V., Speranskaya A. S., Darii M. V., Stepanov O. A., Barkhatov I. M., Kudryavtseva A. V. (2013). Differential Expression of Genes That Encode Glycolysis Enzymes in Kidney and Lung Cancer in Humans. Генетика.

[16] Krasnov GS, Dmitriev AA, Lakunina VA, Kirpiy AA, Kudryavtseva AV (2013). Targeting VDAC-bound hexokinase II: a promising approach for concomitant anti-cancer therapy. Expert Opin Ther Targets.

[17] Krasnov GS, Dmitriev AA, Snezhkina AV, Kudryavtseva AV (2013). Deregulation of glycolysis in cancer: glyceraldehyde-3-phosphate dehydrogenase as a therapeutic target. Expert Opin Ther Targets.

[18] Krasnov GS, Dmitriev AA, Sadtritdinova AF, Fedorova MS, Snezhkina AV, Melnikova NV, Poteryakhina AV, Nyushko KM, Belyakov MM, Kaprin AD, et al. Evaluation of gene expression of hexokinases in colorectal cancer with the use of bioinformatics methods. Biofizika. 2015;60(6):1050–6. 26855992

[19] He X, Lin X, Cai M, Zheng X, Lian L, Fan D, Wu X, Lan P, Wang J (2016). Overexpression of hexokinase 1 as a poor prognosticator in human colorectal cancer. Tumour biology : the journal of the International Society for Oncodevelopmental Biology and Medicine.

[20] Katagiri M, Karasawa H, Takagi K, Nakayama S, Yabuuchi S, Fujishima F, Naitoh T, Watanabe M, Suzuki T, Unno M (2017). Hexokinase 2 in colorectal cancer: a potent prognostic factor associated with glycolysis, proliferation and migration. Histol Histopathol.

[21] Toyota M, Ahuja N, Ohe-Toyota M, Herman JG, Baylin SB, Issa JP (1999). CpG island methylator phenotype in colorectal cancer. Proc Natl Acad Sci U S A.

[22] Bae JM, Kim JH, Kang GH (2013). Epigenetic alterations in colorectal cancer: the CpG island methylator phenotype. Histol Histopathol.

[23] Hinoue T, Weisenberger DJ, Lange CP, Shen H, Byun HM, Van Den Berg D, Malik S, Pan F, Noushmehr H, van Dijk CM (2012). Genome-scale analysis of aberrant DNA methylation in colorectal cancer. Genome Res.

[24] Hawkins N, Norrie M, Cheong K, Mokany E, Ku SL, Meagher A, O'Connor T, Ward R (2002). CpG island methylation in sporadic colorectal cancers and its relationship to microsatellite instability. Gastroenterology.

[25] Ogino S, Kawasaki T, Kirkner GJ, Kraft P, Loda M, Fuchs CS (2007). Evaluation of markers for CpG island methylator phenotype (CIMP) in colorectal cancer by a large population-based sample. The Journal of molecular diagnostics : JMD.

[26] Gallois C, Laurent-Puig P, Taieb J (2016). Methylator phenotype in colorectal cancer: a prognostic factor or not?. Crit Rev Oncol Hematol.

[27] Wong CC, Qian Y, Yu J (2017). Interplay between epigenetics and metabolism in oncogenesis: mechanisms and therapeutic approaches. Oncogene.

[28] Kalady MF, Dejulius KL, Sanchez JA, Jarrar A, Liu X, Manilich E, Skacel M, Church JM (2012). BRAF mutations in colorectal cancer are associated with distinct clinical characteristics and worse prognosis. Dis Colon Rectum.

[29] Issa JP (2004). CpG island methylator phenotype in cancer. Nat Rev Cancer.

[30] Weisenberger DJ, Siegmund KD, Campan M, Young J, Long TI, Faasse MA, Kang GH, Widschwendter M, Weener D, Buchanan D (2006). CpG island methylator phenotype underlies sporadic microsatellite instability and is tightly associated with BRAF mutation in colorectal cancer. Nat Genet.

[31] Turcan S, Rohle D, Goenka A, Walsh LA, Fang F, Yilmaz E, Campos C, Fabius AW, Lu C, Ward PS (2012). IDH1 mutation is sufficient to establish the glioma hypermethylator phenotype. Nature.

[32] Letouze E, Martinelli C, Loriot C, Burnichon N, Abermil N, Ottolenghi C, Janin M, Menara M, Nguyen AT, Benit P (2013). SDH mutations establish a hypermethylator phenotype in paraganglioma. Cancer Cell.

[33] Waterfall JJ, Killian JK, Meltzer PS (2014). The role of mutation of metabolism-related genes in genomic hypermethylation. Biochem Biophys Res Commun.

[34] Love MI, Huber W, Anders S (2014). Moderated estimation of fold change and dispersion for RNA-seq data with DESeq2. Genome Biol.

[35] Yu G, Wang LG, Han Y, He QY (2012). ClusterProfiler: an R package for comparing biological themes among gene clusters. Omics : a journal of integrative biology.

[36] Luo W, Brouwer C (2013). Pathview: an R/Bioconductor package for pathway-based data integration and visualization. Bioinformatics.

[37] Bosman FT, Carneiro F, Hruban RH, Theise ND (2010). WHO classification of Tumours of the digestive system, WHO classification of Tumours, 4th edition, volume 3.

[38] Ma BB, Mo F, Tong JH, Wong A, Wong SC, Ho WM, Wu C, Lam PW, Chan KF, Chan TS (2015). Elucidating the prognostic significance of KRAS, NRAS, BRAF and PIK3CA mutations in Chinese patients with metastatic colorectal cancer. Asia-Pacific journal of clinical oncology.

[39] Boland CR, Thibodeau SN, Hamilton SR, Sidransky D, Eshleman JR, Burt RW, Meltzer SJ, Rodriguez-Bigas MA, Fodde R, Ranzani GN (1998). A National Cancer Institute workshop on microsatellite instability for cancer detection and familial predisposition: development of international criteria for the determination of microsatellite instability in colorectal cancer. Cancer Res.

[40] Drew DA, Nishihara R, Lochhead P, Kuchiba A, Qian ZR, Mima K, Nosho K, Wu K, Wang M, Giovannucci E (2017). A prospective study of smoking and risk of synchronous colorectal cancers. Am J Gastroenterol.

[41] Ogino S, Kawasaki T, Brahmandam M, Cantor M, Kirkner GJ, Spiegelman D, Makrigiorgos GM, Weisenberger DJ, Laird PW, Loda M (2006). Precision and performance characteristics of bisulfite conversion and real-time PCR (MethyLight) for quantitative DNA methylation analysis. The Journal of molecular diagnostics : JMD.

[42] Krasnov GS, Kudryavtseva AV, Snezhkina AV, Lakunina VA, Beniaminov AD, Melnikova NV, Dmitriev AA. Pan-cancer analysis of TCGA data revealed promising reference genes for qPCR normalization. Front Genet. 2019;10:97. 10.3389/fgene.2019.00097 PMC6406071 30881377

[43] Krasnov G. S., Oparina N. Yu., Dmitriev A. A., Kudryavtseva A. V., Anedchenko E. A., Kondrat’eva T. T., Zabarovsky E. R., Senchenko V. N. (2011). RPN1, a new reference gene for quantitative data normalization in lung and kidney cancer. Molecular Biology.

[44] Melnikova NV, Dmitriev AA, Belenikin MS, Koroban NV, Speranskaya AS, Krinitsina AA, Krasnov GS, Lakunina VA, Snezhkina AV, Sadritdinova AF (2016). Identification, expression analysis, and target prediction of flax Genotroph MicroRNAs under Normal and nutrient stress conditions. Front Plant Sci.

[45] Senchenko VN, Anedchenko EA, Kondratieva TT, Krasnov GS, Dmitriev AA, Zabarovska VI, Pavlova TV, Kashuba VI, Lerman MI, Zabarovsky ER (2010). Simultaneous down-regulation of tumor suppressor genes RBSP3/CTDSPL, NPRL2/G21 and RASSF1A in primary non-small cell lung cancer. BMC Cancer.

[46] Pavlova NN, Thompson CB (2016). The emerging hallmarks of Cancer metabolism. Cell Metab.

[47] Guo E, Wei H, Liao X, Xu Y, Li S, Zeng X (2018). Prognostic value of alcohol dehydrogenase mRNA expression in gastric cancer. Oncol Lett.

[48] Ghosh S, Bankura B, Ghosh S, Saha ML, Pattanayak AK, Ghatak S, Guha M, Nachimuthu SK, Panda CK, Maji S (2017). Polymorphisms in ADH1B and ALDH2 genes associated with the increased risk of gastric cancer in West Bengal, India. BMC Cancer.

[49] Ogawa H, Nagano H, Konno M, Eguchi H, Koseki J, Kawamoto K, Nishida N, Colvin H, Tomokuni A, Tomimaru Y (2015). The combination of the expression of hexokinase 2 and pyruvate kinase M2 is a prognostic marker in patients with pancreatic cancer. Molecular and clinical oncology.

[50] Lyshchik A, Higashi T, Hara T, Nakamoto Y, Fujimoto K, Doi R, Imamura M, Saga T, Togashi K (2007). Expression of glucose transporter-1, hexokinase-II, proliferating cell nuclear antigen and survival of patients with pancreatic cancer. Cancer Investig.

[51] Brown RS, Goodman TM, Zasadny KR, Greenson JK, Wahl RL (2002). Expression of hexokinase II and Glut-1 in untreated human breast cancer. Nucl Med Biol.

[52] Lee HG, Kim H, Son T, Jeong Y, Kim SU, Dong SM, Park YN, Lee JD, Lee JM, Park JH (2016). Regulation of HK2 expression through alterations in CpG methylation of the HK2 promoter during progression of hepatocellular carcinoma. Oncotarget.

[53] Mathupala SP, Heese C, Pedersen PL (1997). Glucose catabolism in cancer cells. The type II hexokinase promoter contains functionally active response elements for the tumor suppressor p53. J Biol Chem.

[54] Hamabe A, Yamamoto H, Konno M, Uemura M, Nishimura J, Hata T, Takemasa I, Mizushima T, Nishida N, Kawamoto K (2014). Combined evaluation of hexokinase 2 and phosphorylated pyruvate dehydrogenase-E1alpha in invasive front lesions of colorectal tumors predicts cancer metabolism and patient prognosis. Cancer Sci.

[55] Pudova EA, Kudryavtseva AV, Fedorova MS, Zaretsky AR, Shcherbo DS, Lukyanova EN, Popov AY, Sadritdinova AF, Abramov IS, Kharitonov SL (2018). HK3 overexpression associated with epithelial-mesenchymal transition in colorectal cancer. BMC Genomics.

[56] Hildebrandt T, Knuesting J, Berndt C, Morgan B, Scheibe R (2015). Cytosolic thiol switches regulating basic cellular functions: GAPDH as an information hub?. Biol Chem.

[57] Zhang JY, Zhang F, Hong CQ, Giuliano AE, Cui XJ, Zhou GJ, Zhang GJ, Cui YK (2015). Critical protein GAPDH and its regulatory mechanisms in cancer cells. Cancer biology & medicine.

[58] Khimani AH, Mhashilkar AM, Mikulskis A, O'Malley M, Liao J, Golenko EE, Mayer P, Chada S, Killian JB, Lott ST (2005). Housekeeping genes in cancer: normalization of array data. BioTechniques.

[59] Valenti MT, Bertoldo F, Dalle Carbonare L, Azzarello G, Zenari S, Zanatta M, Balducci E, Vinante O, Lo Cascio V (2006). The effect of bisphosphonates on gene expression: GAPDH as a housekeeping or a new target gene?. BMC Cancer.

[60] Guo C, Liu S, Sun MZ (2013). Novel insight into the role of GAPDH playing in tumor. Clinical & translational oncology : official publication of the Federation of Spanish Oncology Societies and of the National Cancer Institute of Mexico.

[61] Hao L, Zhou X, Liu S, Sun M, Song Y, Du S, Sun B, Guo C, Gong L, Hu J (2015). Elevated GAPDH expression is associated with the proliferation and invasion of lung and esophageal squamous cell carcinomas. Proteomics.

[62] Revillion F, Pawlowski V, Hornez L, Peyrat JP (2000). Glyceraldehyde-3-phosphate dehydrogenase gene expression in human breast cancer. Eur J Cancer.

[63] Jung M, Ramankulov A, Roigas J, Johannsen M, Ringsdorf M, Kristiansen G, Jung K (2007). In search of suitable reference genes for gene expression studies of human renal cell carcinoma by real-time PCR. BMC Mol Biol.

[64] Tokunaga K, Nakamura Y, Sakata K, Fujimori K, Ohkubo M, Sawada K, Sakiyama S (1987). Enhanced expression of a glyceraldehyde-3-phosphate dehydrogenase gene in human lung cancers. Cancer Res.

[65] Ahsan H, Halpern J, Kibriya MG, Pierce BL, Tong L, Gamazon E, McGuire V, Felberg A, Shi J, Jasmine F (2014). A genome-wide association study of early-onset breast cancer identifies PFKM as a novel breast cancer gene and supports a common genetic spectrum for breast cancer at any age. Cancer epidemiology, biomarkers & prevention : a publication of the American Association for Cancer Research, cosponsored by the American Society of Preventive Oncology.

[66] Sun CM, Xiong DB, Yan Y, Geng J, Liu M, Yao XD (2016). Genetic alteration in phosphofructokinase family promotes growth of muscle-invasive bladder cancer. Int J Biol Markers.

[67] Caspi M, Perry G, Skalka N, Meisel S, Firsow A, Amit M, Rosin-Arbesfeld R (2014). Aldolase positively regulates of the canonical Wnt signaling pathway. Mol Cancer.

[68] He J, Jin Y, Chen Y, Yao HB, Xia YJ, Ma YY, Wang W, Shao QS (2016). Downregulation of ALDOB is associated with poor prognosis of patients with gastric cancer. OncoTargets and therapy.

[69] Tao QF, Yuan SX, Yang F, Yang S, Yang Y, Yuan JH, Wang ZG, Xu QG, Lin KY, Cai J (2015). Aldolase B inhibits metastasis through ten-eleven translocation 1 and serves as a prognostic biomarker in hepatocellular carcinoma. Mol Cancer.

[70] Tsai ST, Chien IH, Shen WH, Kuo YZ, Jin YT, Wong TY, Hsiao JR, Wang HP, Shih NY, Wu LW (2010). ENO1, a potential prognostic head and neck cancer marker, promotes transformation partly via chemokine CCL20 induction. Eur J Cancer.

[71] Tu SH, Chang CC, Chen CS, Tam KW, Wang YJ, Lee CH, Lin HW, Cheng TC, Huang CS, Chu JS (2010). Increased expression of enolase alpha in human breast cancer confers tamoxifen resistance in human breast cancer cells. Breast Cancer Res Treat.

[72] Song Y, Luo Q, Long H, Hu Z, Que T, Zhang X, Li Z, Wang G, Yi L, Liu Z (2014). Alpha-enolase as a potential cancer prognostic marker promotes cell growth, migration, and invasion in glioma. Mol Cancer.

[73] Capello M, Ferri-Borgogno S, Riganti C, Chattaragada MS, Principe M, Roux C, Zhou W, Petricoin EF, Cappello P, Novelli F (2016). Targeting the Warburg effect in cancer cells through ENO1 knockdown rescues oxidative phosphorylation and induces growth arrest. Oncotarget.

[74] Schneider CC, Archid R, Fischer N, Buhler S, Venturelli S, Berger A, Burkard M, Kirschniak A, Bachmann R, Konigsrainer A (2015). Metabolic alteration--overcoming therapy resistance in gastric cancer via PGK-1 inhibition in a combined therapy with standard chemotherapeutics. Int J Surg.

[75] Zieker D, Konigsrainer I, Weinreich J, Beckert S, Glatzle J, Nieselt K, Buhler S, Loffler M, Gaedcke J, Northoff H (2010). Phosphoglycerate kinase 1 promoting tumor progression and metastasis in gastric cancer - detected in a tumor mouse model using positron emission tomography/magnetic resonance imaging. Cellular physiology and biochemistry : international journal of experimental cellular physiology, biochemistry, and pharmacology.

[76] Zhang D, Tai LK, Wong LL, Chiu LL, Sethi SK, Koay ES (2005). Proteomic study reveals that proteins involved in metabolic and detoxification pathways are highly expressed in HER-2/neu-positive breast cancer. Molecular & cellular proteomics : MCP.

[77] Wang J, Wang J, Dai J, Jung Y, Wei CL, Wang Y, Havens AM, Hogg PJ, Keller ET, Pienta KJ (2007). A glycolytic mechanism regulating an angiogenic switch in prostate cancer. Cancer Res.

[78] Hwang TL, Liang Y, Chien KY, Yu JS (2006). Overexpression and elevated serum levels of phosphoglycerate kinase 1 in pancreatic ductal adenocarcinoma. Proteomics.

[79] Duan Z, Lamendola DE, Yusuf RZ, Penson RT, Preffer FI, Seiden MV (2002). Overexpression of human phosphoglycerate kinase 1 (PGK1) induces a multidrug resistance phenotype. Anticancer Res.

[80] Shashni B, Sakharkar KR, Nagasaki Y, Sakharkar MK (2013). Glycolytic enzymes PGK1 and PKM2 as novel transcriptional targets of PPARgamma in breast cancer pathophysiology. J Drug Target.

[81] Cui J, Quan M, Jiang W, Hu H, Jiao F, Li N, Jin Z, Wang L, Wang Y, Wang L (2015). Suppressed expression of LDHB promotes pancreatic cancer progression via inducing glycolytic phenotype. Med Oncol.

[82] Leiblich A, Cross SS, Catto JW, Phillips JT, Leung HY, Hamdy FC, Rehman I (2006). Lactate dehydrogenase-B is silenced by promoter hypermethylation in human prostate cancer. Oncogene.

[83] Maekawa M, Taniguchi T, Ishikawa J, Sugimura H, Sugano K, Kanno T (2003). Promoter hypermethylation in cancer silences LDHB, eliminating lactate dehydrogenase isoenzymes 1-4. Clin Chem.

[84] McCleland ML, Adler AS, Shang Y, Hunsaker T, Truong T, Peterson D, Torres E, Li L, Haley B, Stephan JP (2012). An integrated genomic screen identifies LDHB as an essential gene for triple-negative breast cancer. Cancer Res.

[85] Shi H, Fang R, Li Y, Li L, Zhang W, Wang H, Chen F, Zhang S, Zhang X, Ye L (2016). The oncoprotein HBXIP suppresses gluconeogenesis through modulating PCK1 to enhance the growth of hepatoma cells. Cancer Lett.

[86] Wang B, Hsu SH, Frankel W, Ghoshal K, Jacob ST (2012). Stat3-mediated activation of microRNA-23a suppresses gluconeogenesis in hepatocellular carcinoma by down-regulating glucose-6-phosphatase and peroxisome proliferator-activated receptor gamma, coactivator 1 alpha. Hepatology.

[87] Wang P, Mai C, Wei YL, Zhao JJ, Hu YM, Zeng ZL, Yang J, Lu WH, Xu RH, Huang P (2013). Decreased expression of the mitochondrial metabolic enzyme aconitase (ACO2) is associated with poor prognosis in gastric cancer. Med Oncol.

[88] Santos CR, Schulze A (2012). Lipid metabolism in cancer. FEBS J.

[89] Hanai JI, Doro N, Seth P, Sukhatme VP (2013). ATP citrate lyase knockdown impacts cancer stem cells in vitro. Cell Death Dis.

[90] Lee S, Nakamura E, Yang H, Wei W, Linggi MS, Sajan MP, Farese RV, Freeman RS, Carter BD, Kaelin WG (2005). Neuronal apoptosis linked to EglN3 prolyl hydroxylase and familial pheochromocytoma genes: developmental culling and cancer. Cancer Cell.

[91] Selak MA, Armour SM, MacKenzie ED, Boulahbel H, Watson DG, Mansfield KD, Pan Y, Simon MC, Thompson CB, Gottlieb E (2005). Succinate links TCA cycle dysfunction to oncogenesis by inhibiting HIF-alpha prolyl hydroxylase. Cancer Cell.

[92] Desideri E, Vegliante R, Ciriolo MR (2015). Cancer letters.

[93] Gao P, Tchernyshyov I, Chang TC, Lee YS, Kita K, Ochi T, Zeller KI, De Marzo AM, Van Eyk JE, Mendell JT (2009). C-Myc suppression of miR-23a/b enhances mitochondrial glutaminase expression and glutamine metabolism. Nature.

[94] Wise DR, Thompson CB (2010). Glutamine addiction: a new therapeutic target in cancer. Trends Biochem Sci.

[95] Mullen AR, Hu Z, Shi X, Jiang L, Boroughs LK, Kovacs Z, Boriack R, Rakheja D, Sullivan LB, Linehan WM (2014). Oxidation of alpha-ketoglutarate is required for reductive carboxylation in cancer cells with mitochondrial defects. Cell Rep.

[96] Anderson NM, Mucka P, Kern JG, Feng H (2018). The emerging role and targetability of the TCA cycle in cancer metabolism. Protein & cell.

[97] Li WL, Xiao MS, Zhang DF, Yu D, Yang RX, Li XY, Yao YG (2014). Mutation and expression analysis of the IDH1, IDH2, DNMT3A, and MYD88 genes in colorectal cancer. Gene.

[98] Stachler MD, Rinehart E, Lindeman N, Odze R, Srivastava A (2015). Novel molecular insights from routine genotyping of colorectal carcinomas. Hum Pathol.

[99] M Gagné L, Boulay K, Topisirovic I, Huot ME, Mallette FA (2017). Oncogenic activities of IDH1/2 mutations: from epigenetics to cellular signaling. Trends Cell Biol.

[100] Reitman ZJ, Yan H (2010). Isocitrate dehydrogenase 1 and 2 mutations in cancer: alterations at a crossroads of cellular metabolism. J Natl Cancer Inst.

[101] Sanchez-Vega F, Gotea V, Chen YC, Elnitski L (2017). CpG island methylator phenotype in adenocarcinomas from the digestive tract: methods, conclusions, and controversies. World journal of gastrointestinal oncology.

[102] Vedeld HM, Merok M, Jeanmougin M, Danielsen SA, Honne H, Presthus GK, Svindland A, Sjo OH, Hektoen M, Eknaes M (2017). CpG island methylator phenotype identifies high risk patients among microsatellite stable BRAF mutated colorectal cancers. Int J Cancer.

[103] Durany N, Joseph J, Jimenez OM, Climent F, Fernandez PL, Rivera F, Carreras J (2000). Phosphoglycerate mutase, 2,3-bisphosphoglycerate phosphatase, creatine kinase and enolase activity and isoenzymes in breast carcinoma. Br J Cancer.

[104] Schmechel D, Marangos PJ, Brightman M (1978). Neurone-specific enolase is a molecular marker for peripheral and central neuroendocrine cells. Nature.

[105] Yeh CS, Wang JY, Chung FY, Lee SC, Huang MY, Kuo CW, Yang MJ, Lin SR (2008). Significance of the glycolytic pathway and glycolysis related-genes in tumorigenesis of human colorectal cancers. Oncol Rep.

[106] Jogi A, Vaapil M, Johansson M, Pahlman S (2012). Cancer cell differentiation heterogeneity and aggressive behavior in solid tumors. Ups J Med Sci.

[107] Pancholi V (2001). Multifunctional alpha-enolase: its role in diseases. Cellular and molecular life sciences : CMLS.

[108] Selga E, Morales C, Noe V, Peinado MA, Ciudad CJ (2008). Role of caveolin 1, E-cadherin, enolase 2 and PKCalpha on resistance to methotrexate in human HT29 colon cancer cells. BMC Med Genet.

[109] Ledermann JA (1994). Serum neurone-specific enolase and other neuroendocrine markers in lung cancer. Eur J Cancer.

[110] Sun L, Guo C, Cao J, Burnett J, Yang Z, Ran Y, Sun D (2017). Over-expression of alpha-enolase as a prognostic biomarker in patients with pancreatic Cancer. Int J Med Sci.

[111] Soh MA, Garrett SH, Somji S, Dunlevy JR, Zhou XD, Sens MA, Bathula CS, Allen C, Sens DA (2011). Arsenic, cadmium and neuron specific enolase (ENO2, gamma-enolase) expression in breast cancer. Cancer Cell Int.

[112] Kim J, Jin H, Zhao JC, Yang YA, Li Y, Yang X, Dong X, Yu J (2017). FOXA1 inhibits prostate cancer neuroendocrine differentiation. Oncogene.

[113] Nguyen A, Loo JM, Mital R, Weinberg EM, Man FY, Zeng Z, Paty PB, Saltz L, Janjigian YY, de Stanchina E (2016). PKLR promotes colorectal cancer liver colonization through induction of glutathione synthesis. J Clin Invest.

[114] Korsmeyer SJ, Wei MC, Saito M, Weiler S, Oh KJ, Schlesinger PH (2000). Pro-apoptotic cascade activates BID, which oligomerizes BAK or BAX into pores that result in the release of cytochrome c. Cell Death Differ.

[115] Shoshan-Barmatz V, Mizrachi D (2012). VDAC1: from structure to cancer therapy. Front Oncol.

[116] Chiara F, Castellaro D, Marin O, Petronilli V, Brusilow WS, Juhaszova M, Sollott SJ, Forte M, Bernardi P, Rasola A (2008). Hexokinase II detachment from mitochondria triggers apoptosis through the permeability transition pore independent of voltage-dependent anion channels. PLoS One.

[117] Wu CC, Bratton SB (2013). Regulation of the intrinsic apoptosis pathway by reactive oxygen species. Antioxid Redox Signal.

[118] Starkov AA, Fiskum G, Chinopoulos C, Lorenzo BJ, Browne SE, Patel MS, Beal MF (2004). Mitochondrial alpha-ketoglutarate dehydrogenase complex generates reactive oxygen species. J Neurosci.

[119] Sen T, Sen N, Noordhuis MG, Ravi R, Wu TC, Ha PK, Sidransky D, Hoque MO (2012). OGDHL is a modifier of AKT-dependent signaling and NF-kappaB function. PLoS One.

