# Supplementary figures and images for: The CIMP-high phenotype is associated with energy metabolism alterations in colon adenocarcinoma
Source: BMC Med Genet. 2019 Apr 9;20(Suppl 1):52. doi: 10.1186/s12881-019-0771-5 (PMC6454590; doi:10.1186/s12881-019-0771-5)

# GLYCOLYSIS / GLUCONEOGENESIS

Relative scale

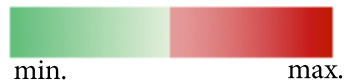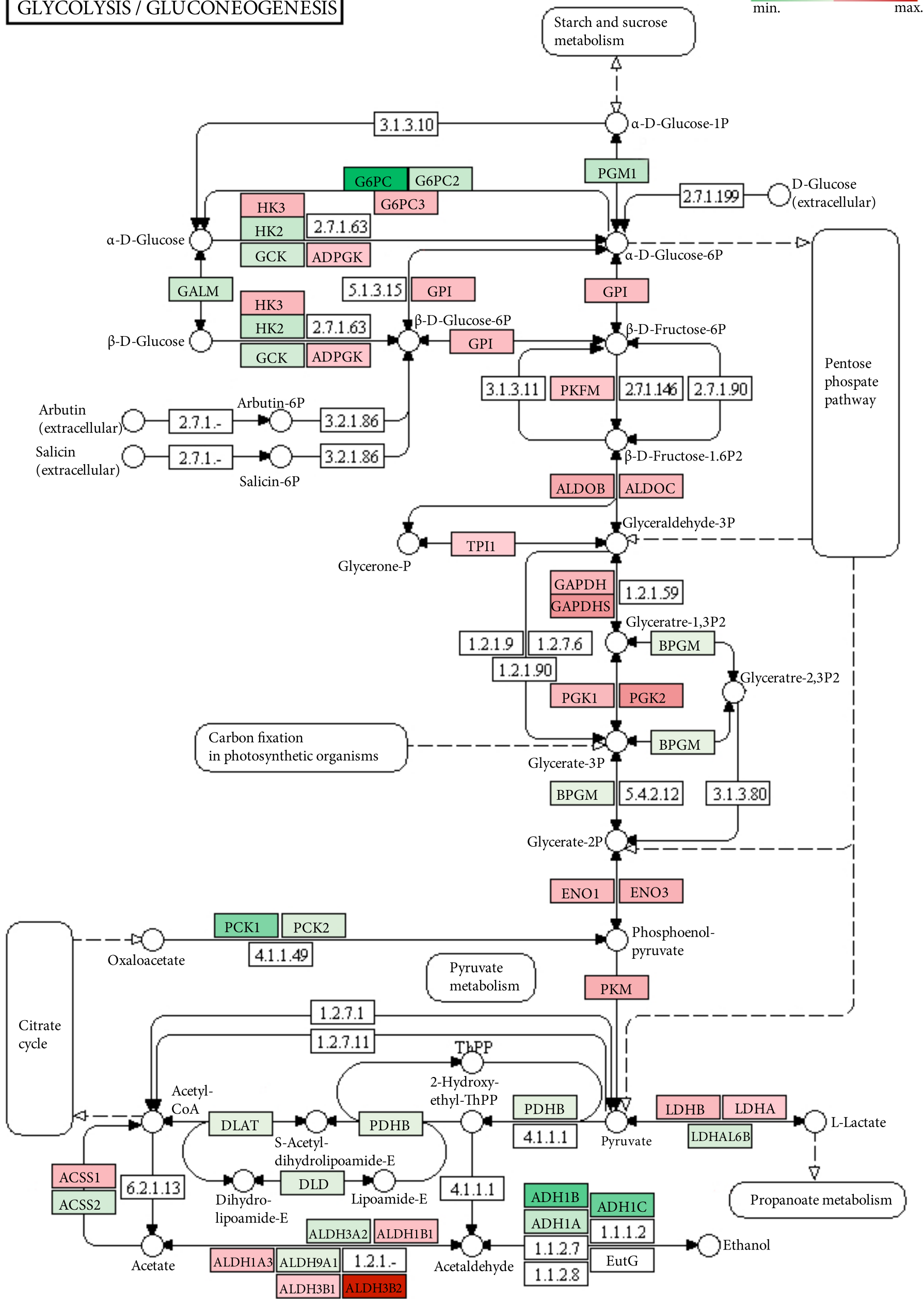

Supplement: Supplementary file 1 — Figure S1. Differential expression profiles of “Glycolysis/Gluconeogenesis” KEGG pathway genes in colon adenocarcinomas compared to normal tissues (TCGA data). The color scale reflects the base-2 logarithm of the ratio of the gene expression level in tumor tissues to the expression level in normal tissues. Red color indicates upregulated genes, green - downregulated. The scale is relative; minimal and maximal values in the color scale are corresponding to the greatest decreased or increased expression level among the all analyzed genes. (PDF 5115 kb) [file 12881_2019_771_MOESM1_ESM.pdf]

# CITRATE CYCLE (TCA CYCLE)

Relative scale

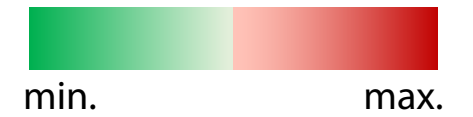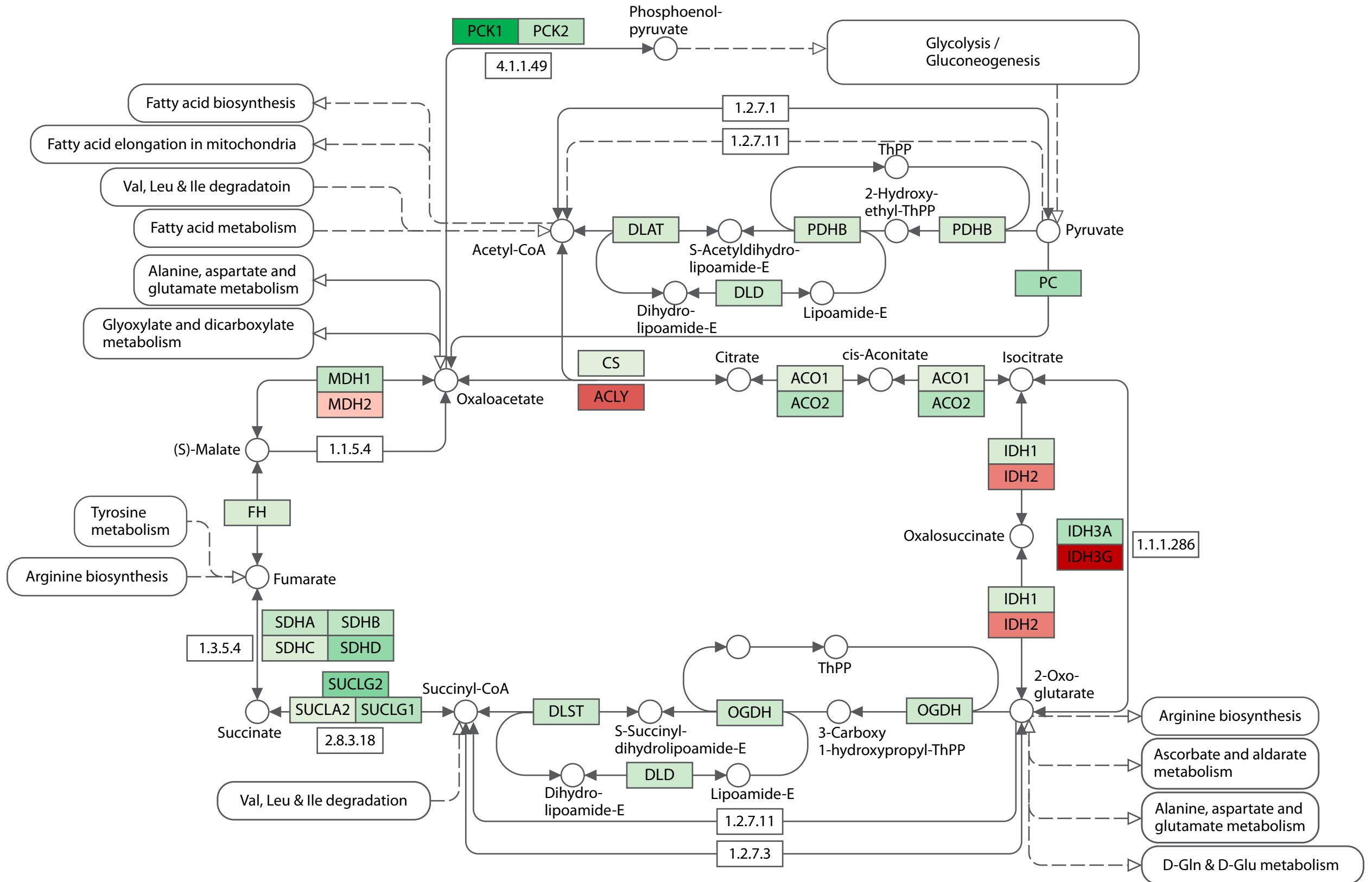

Data on KEGG graph

Supplement: Supplementary file 3 — Figure S2. Differential expression profiles of “TCA cycle” KEGG pathway genes in colon adenocarcinomas compared to normal tissues (TCGA data). The color scale reflects the base-2 logarithm of the ratio of the gene expression level in tumor tissues to the expression level in normal tissues. Red color indicates upregulated genes, green - downregulated. The scale is relative; minimal and maximal values in the color scale are corresponding to the greatest decreased or increased expression level among the all analyzed genes. (PDF 173 kb) [file 12881_2019_771_MOESM3_ESM.pdf]

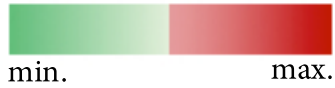

GLYCOLYSIS / GLUCONEOGENESIS

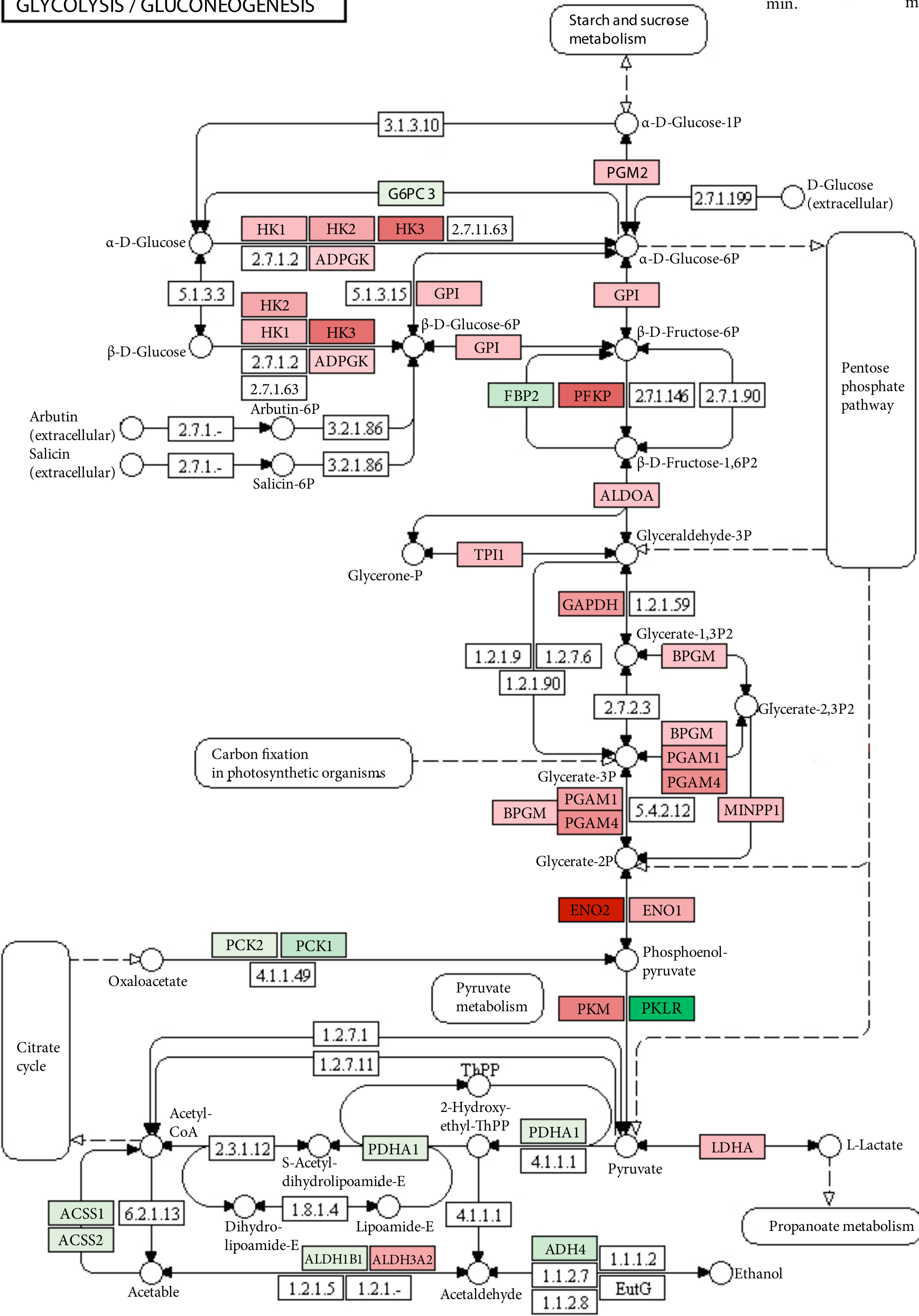

Supplement: Supplementary file 5 — Figure S3. Differential expression profiles of “Glycolysis/Gluconeogenesis” KEGG pathway genes in CIMP-high colon adenocarcinomas compared to non-CIMP ones (TCGA data). The color scale reflects the base-2 logarithm of the ratio of the gene expression level in CIMP-high tumors to the expression level in non-CIMP tumors. Red color indicates upregulated genes, green - downregulated. The scale is relative; minimal and maximal values in the color scale are corresponding to the greatest decreased or increased expression level among the all analyzed genes. (PDF 4613 kb) [file 12881_2019_771_MOESM5_ESM.pdf]

CITRATE CYCLE (TCA CYCLE)

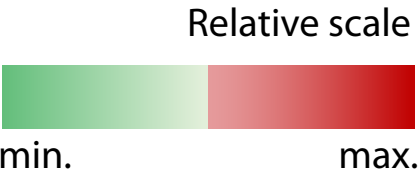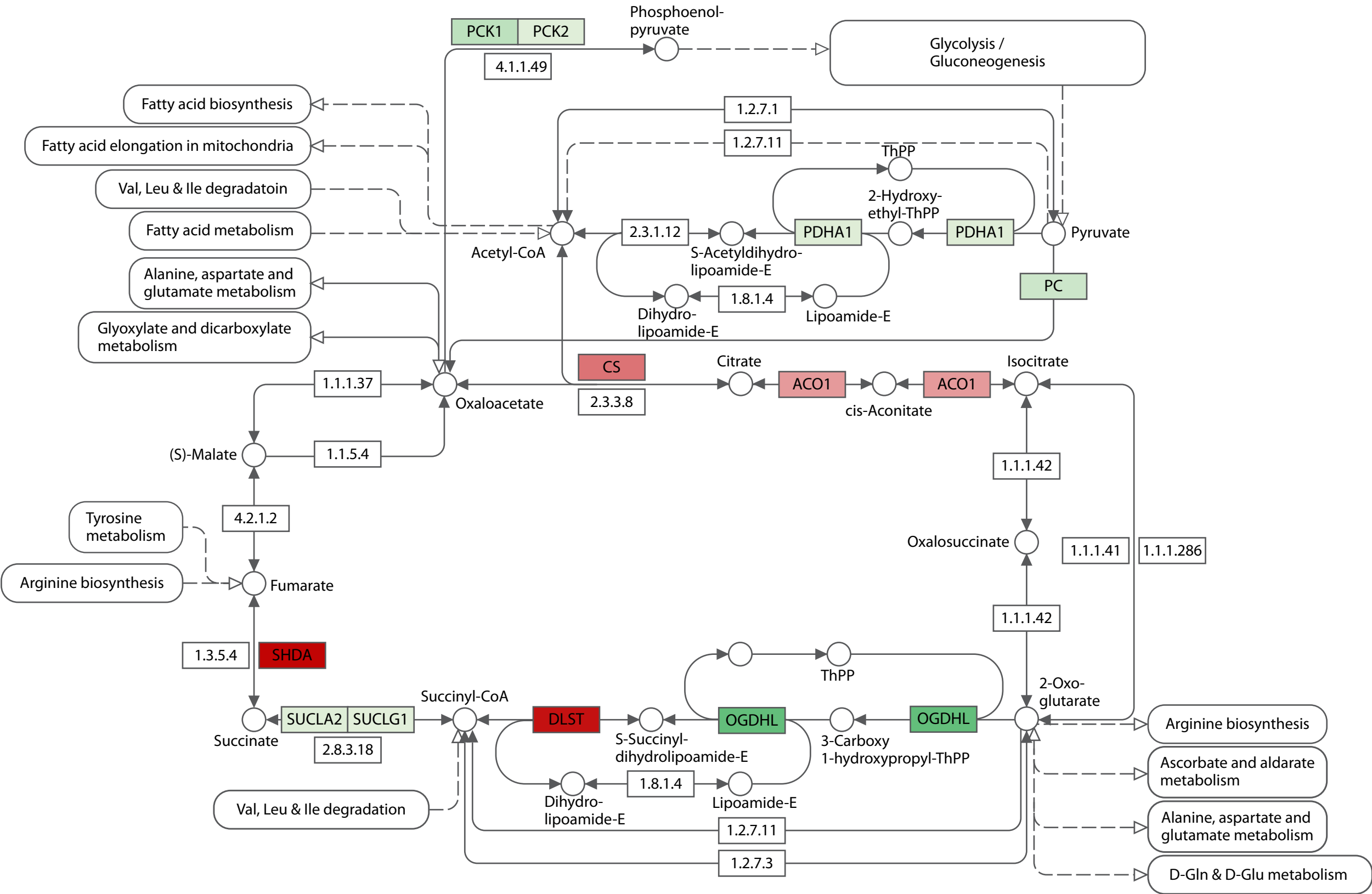

Data on KEGG graph

Supplement: Supplementary file 7 — Figure S4. Differential expression profiles of “TCA cycle” KEGG pathway genes in CIMP-high colon adenocarcinomas compared to non-CIMP (TCGA data). The color scale reflects the base-2 logarithm of the ratio of the gene expression level in CIMP-high tumors to the expression level in non-CIMP tumors. Red color indicates upregulated genes, green - downregulated. The scale is relative; minimal and maximal values in the color scale are corresponding to the greatest decreased or increased expression level among the all analyzed genes. (PDF 928 kb) [file 12881_2019_771_MOESM7_ESM.pdf]
